# Label-Free Direct Detection of Cylindrospermopsin via Graphene-Enhanced Surface Plasmon Resonance Aptasensor

**DOI:** 10.3390/toxins15050326

**Published:** 2023-05-10

**Authors:** Stefan Jaric, Aabha Bajaj, Vladimir Vukic, Ivana Gadjanski, Ibrahim Abdulhalim, Ivan Bobrinetskiy

**Affiliations:** 1 BioSense Institute-Research and Development Institute for Information Technologies in Biosystems, University of Novi Sad, 21000 Novi Sad, Serbia; igadjanski@biosense.rs; 2 Department of Electro-Optics and Photonics Engineering, School of Electrical and Computer Engineering, Ilse-Katz Institute for Nano-Scale Science and Technology, Ben Gurion University, Beer Sheva 84105, Israel; bajaj@post.bgu.ac.il (A.B.); abdulhlm@bgu.ac.il (I.A.); 3 Faculty of Technology Novi Sad, University of Novi Sad, Bulevar Cara Lazara 1, 21000 Novi Sad, Serbia; 4 Photonicsys Ltd., 54 Wahat Alsalam-Neveh Shalom, Ibrahim 9976100, Israel

**Keywords:** CVD graphene, label-free biosensing, cyanotoxins, aptamer, surface plasmon resonance

## Abstract

In this work, we report a novel method for the label-free detection of cyanotoxin molecules based on a direct assay utilizing a graphene-modified surface plasmon resonance (SPR) aptasensor. Molecular dynamic simulation of the aptamer’s interaction with cylindrospermopsin (CYN) reveals the strongest binding sites between C18–C26 pairs. To modify the SPR sensor, the wet transfer method of CVD monolayer graphene was used. For the first time, we report the use of graphene functionalized by an aptamer as a bioreceptor in conjunction with SPR for the detection of CYN. In a direct assay with an anti-CYN aptamer, we demonstrated a noticeable change in the optical signal in response to the concentrations far below the maximum tolerable level of 1 µg/L and high specificity.

## 1. Introduction

Algal blooms are a common issue in almost all kinds of freshwater or marine water systems. Global warming and the excessive nutrient richness caused by wastewater, air pollution and fertilizers has increased the number of water sources suffering from algal blooms. Recent global research has revealed that about 9% of freshwater lakes across six continents have experienced algal blooms [1]. Many Cyanobacteria (blue-green algae) produce dangerous and very stable toxins (cyanotoxins), which can pollute the water systems [2]. The contamination of raw water can limit its suitability for many purposes. The purification of the contaminated water might be quite expensive, which makes continuous and large-scale treatment economically unfeasible in many cases [3] that require separation for the analysis of the degree of water toxicity [4].

Water quality control is managed by strong legislation in many regions [5]. Nevertheless, the rising amount of processed water, including water storage, recycling and transfer chains, increases the risk of distributing cyanotoxins to end users. Different methods are used for accurate water quality control based on the detection of bacterial contamination (e.g., polymerase chain reaction (PCR)) or direct measurements of toxins using ELISA (enzyme-linked immunosorbent assay) or RPLA (reversed passive latex agglutination) tests [6]. For quality control in the production and post-production chains in aquaculture, more robust and durable technologies need to be applied. Surface plasmon resonance (SPR) is a well-established technique that has been used to detect a wide range of analytes. Moreover, it can be integrated with advanced methods such as continuous PCR analysis [7,8], turning SPR into a universal platform for real-time diagnostics. Recently, SPR has been used for the detection of cyanotoxins generated by algal blooms [9]. The technique demonstrated less sensitivity to such small molecules than a standard ELISA detection [10], hence additional SPR signal amplification methods must be developed [11]. In fact, there is a need for a highly sensitive direct assay for in-field rapid diagnostics of water quality. A graphene monolayer in the SPR biosensor can remarkably increase SPR sensitivity by up to 3.5 times compared to gold-based SPR with demonstrated sensitivity up to 6.4 nm/% [12,13]. The physics behind this enhancement can be understood as a consequence of the large refractive index of graphene, as it was shown in many works that such a high-index top nanolayer increases the sensitivity due to the increase in the field overlap integral [14,15,16,17]. In particular, the figure of merit in the spectral mode was found to increase. Moreover, graphene can provide higher-density packages for the binding of bioreceptors to the surface.

In this work, we have developed an SPR aptasensor modified with graphene for the detection of one type of cyanotoxin-cylindrospermopsin (CYN) produced by various freshwater Cyanobacteria. The mechanism of detection is based on the changes in anti-CYN aptamer conformation upon binding of the analyte. Such sensors allow, with the required accuracy and in a short time, small concentrations of cyanotoxins released by Cyanobacteria to be detected in freshwater.

## 2. Results and Discussion

The SPR chips used in this work are from Photonicsys Ltd. (Neveh Shalom, Israel), based on SF11 glass and covered by Au/Ti (50/2 nm) film, and have been demonstrated previously to be a suitable substrate for DNA analysis [18]. The shift of the resonant spectrum of the SPR chip through the spectrometer is actively used for analyte quantification due to its high accuracy [19]. Spectral mode SPR facilities of Photonicsys Ltd., (www.photonicsys.com, accessed on 1 May 2023) were used. In order to increase the sensitivity of the optical method, we used a recently suggested approach based on graphene-enhanced optical sensors [20]. The full fabrication overview is given in the Materials and Methods section. An aptamer was used as a bioreceptor to bind cyanotoxin molecules.

**Figure 1:**
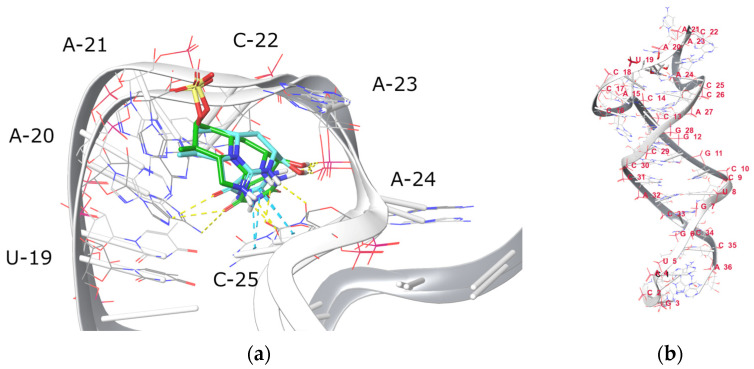
Structure of the 36 nucleotides truncated aptamer (CY9T2): (**a**) overlapped structures of CY9T2 in the complex with CYN before (green) and after the MD simulation (cyan); yellow lines—polar interactions, cyan lines—π-π stacking interactions; (**b**) structure of the CY9T2 aptamer.

Aptamers are being increasingly used as bioreceptors for toxin detection due to their high sensitivity and ease of fabrication, particularly in comparison to antibodies which are very difficult to produce for toxin recognition. Still, additional efforts are needed to address proper selectivity, which can be effectively achieved using in silico methods [21]. The molecular docking method followed by molecular dynamics (MD) simulations were used to indicate potential binding sites of CYN with aptamers, described in detail in [22]. In the stated research, C1–T5 bases of the CY9T2 aptamer were proposed to be involved in the binding of the CYN. According to our modelling results, the optimized (CY9T2) aptamer may have an additional binding site for CYN. In the CY9 aptamer, CYN is wrapped in the chains between C33–C41 and G21–C24, and during the MD simulation, strongly interacts with the bases C33–C41. C33–C41 bases are also present in the CY9T2 optimized aptamer and have numbers C18–C26. Docking the CYN with the optimized aptamer CY9T2 in the way that CYN interacts with the C18–C26 bases that are positioned in the loop of the aptamer indicated strong binding. MD simulation of this complex revealed a stable complex, with RMSD of 1.827 Å in the binding site (Figure 1a). During the MD simulation, CYN was interacting with the C25 and C22 as well as the A21 and A23 bases, which corresponds to C40, C37, A36 and A38 bases in the CY9 aptamer. These results indicate the possibility of CYN to bind CY9 and CY9T2 in the proposed binding site. The 3D structure of the CY9T2 aptamer is quite rigid and the C1–T5 bases are at the opposite side compared to the A21–C25 bases and there is no possibility for interaction (Figure 1b). In our research, where we used the amino-modified 5′ end at the site of C1–T5, the only possible binding region is C18–C26.

**Figure 2:**
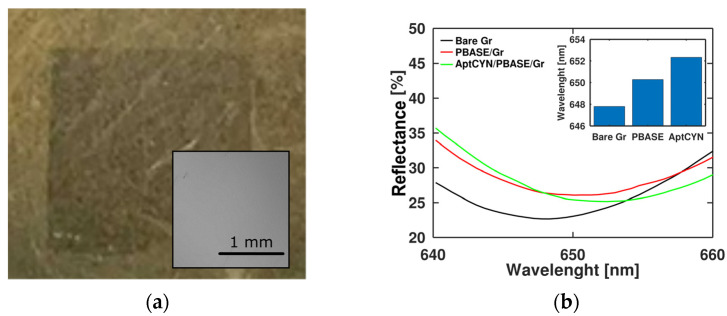
Preparation and assembly of graphene aptasensor on SPR chip: (**a**) optical image of graphene flake after wet transfer onto SPR chip (the physical contrast enhancement was used, see supporting information), inset: SEM image of Gr/SPR; (**b**) optical reflection response of bare Gr/SPR chips after PBASE deposition and aptamer immobilization; inset: Gr/SPR sensor wavelength shift during assembly.

The bare Gr/SPR chip (SPR chips covered by graphene) (see Figure 2a) was modified according to the standard procedure for graphene aptasensor assembly [23]. We used the CY9T2 aptamer for biosensor development. To enhance the graphene image contrast on the gold surface, we have developed a novel physical technique for its visualization (Figure S1, see supporting information). SEM and EDX analysis (Figures S2 and S3, see supporting information) demonstrated the absence of large impurities after graphene transfer. The spectral curves after PBASE deposition and aptamer immobilization were recorded for Gr/SPR reflectance (Figure 2b). Notably, the red shift of about 2 nm of the signal was observed for both molecules. When attached to graphene, both the PBASE and anti-CYN aptamers increased the effective thickness and transferred charge to the surface, which can affect the SPR signal [24]. Atomic force microscopy was used to confirm the integrity of the graphene film transferred onto gold (Figure S4, see supporting information).

**Figure 3:**
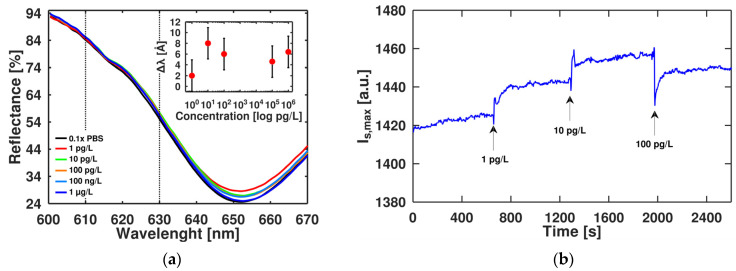
Sensor response to the binding of CYN with aptamer immobilized on Gr/SPR surface: (**a**) SPR reflection curve for Gr/SPR aptasensor at different CYN concentrations (inset: wavelength shift on interaction with different CYN concentrations); (**b**) time evolution of SPR curve’s signal at maximal slope (I_s,max_) change in the range of 610–630 nm during increase in the CYN concentration.

The spectral characteristics of the Gr/SPR chip were measured in the presence of different concentrations of CYN in 0.1 × PBS solution. A dissolved buffer (~15 mM) was chosen to address the ionic strength of freshwater (5–20 mM) more accurately. We observed spectral shifts towards higher wavelengths for increased CYN concentration with saturation at 100 pg/L (Figure 3a) with a maximal shift of 0.8 nm. Still, the spectral resolution degrades due to the spectral broadening [13], which may decrease the sensitivity. Therefore, we switched to monitoring the signal at the maximal slope of the spectral curve in the linear range, which can be more accurate for the real-time monitoring of the aptasensor’s response to low concentration changes of small molecules (Figure 3b) because the rising edge of the SPR curve is much sharper than at the minimum. To improve the signal-to-noise ratio, the integral of the signal between 610 and 630 nm was used as the monitoring parameter (I_s,max_). We observed a decrease in the signal during the exchange of solutions, associated with the dynamic release of molecules. The high affinity of the aptamer to CYN results in a decrease in π-π stacking interactions between the aptamer and graphene [22,25], thus increasing the effective thickness of the aptamer layer. Nevertheless, the high roughness of the substrate that exceeds the Debye length can lead to interference of neighbouring aptamer molecules and decrease the signal while increasing the concentration of the analyte during its accumulation. The roughness effect on binding sites of the aptamer can involve less preferable sites of CY9T2. As a result, the conformational change in the aptamer can be destroyed, and effective thickness can be decreased, leading to signal distortion during the accumulation of CYN. We can estimate the limit of detection based on accumulations of CYN (there are no washing steps between different concentrations) at the level of 100 pg/L.

**Figure 4:**
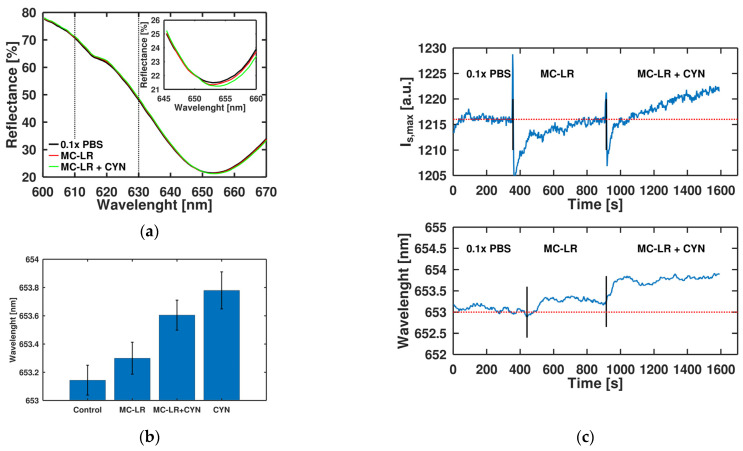
Specificity and cross-selectivity of Gr/SPR aptasensor toward CYN and MC-LR (non-specific target): (**a**) optical reflection response of Gr/SPR chips with varying analytes; (**b**) Gr/SPR sensor response from specific (CYN) and non-specific (MC-LR) targets in concentrations of both 1 µg/L and the mixture of 1 µg/L of MC-LR with 0.1 µg/L of CYN (N = 2); (**c**) time evolution of I_s,max_ change (**top**) and wavelength shift (**bottom**) for consequential replacement of 0.1 × PBS with 1 µg/mL solution of MC-LR and its mixture with 0.1 µg/mL of CYN. The baseline shows the sensor response in buffer after signal saturation.

The selectivity of the Gr/SPR aptasensor was estimated in the same experimental conditions using Microcystin-LR (MC-LR) solution in 0.1 × PBS for concentration 1µg/L, and for the cross-selectivity test we mixed it with 100 ng/L of CYN (Figure 4). The sensor response for MC-LR was on the noise level, while for the CYN with MC-LR mixture we observed a noticeable shift of 0.6 nm (Figure 4a). Notably, adding concentrations of CYN at the same level as MC-LR increases the sensor response (Figure 4b). Real-time experiments for selectivity demonstrate similar trends and I_s_,_max_ changes are more accurate than wavelength shift (Figure 4c). After regeneration of the sensor’s chip, we observed similar trends on repeated experiments but with less specificity (Figure S5, see supporting information). Degradation of the sensor’s characteristics can be related to the high roughness of the gold surface (Figure S4), which weakens the immobilization of the receptor layer. We observed that the response level when starting with a higher concentration of CYN (100 ng/L) is the same level as for lower concentrations without accumulation. This confirms our assumption that a limited number of binding sites results in the low dynamic range of the sensor. Thus, the suggested sensor can be applied for the low-concentration detection of small molecules, while adjusting for the flatness of the gold film needs to be performed to improve the dynamic range.

Cyanotoxins are small molecules with low molecular weight, e.g., for CYN this is 415 Da [26], that can hardly be detected without signal amplification. The enhancement of the sensitivity of SPR-based biosensors is discussed in terms of the synergetic effect of graphene resulting in charge transfer from graphene to gold film and the increased adsorption efficiency [27,28]. The charge couples provide larger dipoles on the graphene–gold interface resulting in higher sensitivity to small changes in analyte layer thickness. We suggest that the conformational change of the aptamer leads to detachment of the aptamer from the graphene surface, thus increasing the effective thickness of the aptamer layer. Recently, it was demonstrated that the use of graphene gradually increases the sensitivity of photoelectrochemical sensors to another cyanotoxin (MC-LR) [29]. Unique optical and electrical properties of graphene can provide amplification of the sensing signal of the SPR aptasensor. We assume that the effect of the aptamer’s conformational change close to the graphene surface [25] can bring negative charge to the surface [30], which in turn affects the Fermi energy level of graphene, which can modulate both real and imaginary parts of the refractive index [31]. Thus, the sensor’s performance can be actively increased in response to the binding of small molecules [13]. A proper choice of aptamer is also important for improving sensitivity. This provides the Gr/SPR with sufficient sensitivity (Table 1), but still with a more significant level of noise that decreases the dynamic range.

## 3. Conclusions

This work reports on the development of a graphene-enhanced surface plasmon resonance aptasensor for the detection of cylindrospermopsin cyanotoxin. We have demonstrated the enhancement of SPR sensitivity towards small molecules by properly adjusting experimental parameters: (i) signal amplification of graphene-modified SPR chips; (ii) accuracy improvement by using resonant SPR spectrum processing; (iii) modeling of aptamer binding to CYN; and (iv) properly designed setup with a highly sensitive spectrometer and collimated light source.

A relatively simple and cost-effective approach based on CVD graphene transfer and aptamer immobilization was suggested to be compatible with the standard SPR technique. MD simulation was used to define the binding sites of CYN and the aptamer. The fabricated aptasensor showed the capability to detect trace levels of CYN concentrations down to 100 pg/L in a solution with ionic strength corresponding to freshwater sources. Regeneration is performed by simply soaking the chip in urea solution. This simple step enables in-field use of the developed biosensor, as the same chip can be used repeatedly after regeneration. This work shows the proof of concept for the general ability of a graphene-based direct assay SPR for cyanotoxin detection in freshwater. Nevertheless, optimizing the noise level and increasing the selectivity is needed to foresee the real-time small molecule detection by SPR methods.

## 4. Materials and Methods

### 4.1. Molecular Modeling Simulations

Structures of CY9 and CY9T2 aptamers were determined and constructed using an RNA fold server (http://rna.tbi.univie.ac.at, accessed on 19 April 2023) and RNA composer server (https://rnacomposer.cs.put.poznan.pl, accessed on 19 April 2023).

All simulations were performed using the Schrödinger package [36] and OPLS4 force field [37].

The 3D structures of CYN were constructed using Maestro software in the Schrödinger software [38]. The geometry optimization of CYN was performed using the OPLS4 force field. Powell conjugated gradient algorithm method was applied with a convergence criterion of 0.01 kcal/(mol Å) and maximum iterations of 1000.

For molecular docking simulations, the programme Glide was used [39]. The following parameters were used in the molecular docking simulations: flexible ligand, extra precision mode and the Epik state penalties. To calculate ligand binding affinities, the MM-GBSA method was performed using the VSGB 2.0 solvation model [40]. In the calculations of MM-GBSA, residues within a distance of 8.0 Å from the ligand were assigned as flexible.

To analyse the obtained docking poses and evaluate their behaviour over time, we performed MD simulations using Desmond software [41]. To construct a system and ensure charge neutrality, water molecules (i.e., TIP3P water model) and ions (Na^+^ and Cl^−^) were added to each system consisting of a ligand and an aptamer. MD simulations were performed using the OPLS4 force field and constant NPT conditions (*p* = 1 atm and T = 298 K). The trajectories were performed for 200 ns. To ensure NPT conditions, the Nose–Hoover chain was performed as thermostat method (relaxation time was 1 ps) and the isotropic Martyna–Tobias–Klein was performed as barostat method (relaxation time was 2 ps). Lennard-Jones and electrostatic interactions were taken into account with the cutoff radius of 9.0 Å. The molecular dynamics time step was 1 fs.

Obtained results were visualized using the Maestro program [38].

### 4.2. Spectral SPR Set Up

For measurements, we used a self-made SPR spectral interrogation system that employs a collimated white light and measures coupling strength across a range of several wavelengths to sense the change in R.I at the point of strongest coupling wavelength, i.e., minimum reflectance. A detailed description of the setup is provided in our previous work [18]. The collimated visible-NIR range beam was passed through a right-angled SF11 prism to excite surface plasmons in the SPR substrate. Index matching oil was used to assemble SPR substrate onto the prism. The reflected light was focused by an objective lens to another fibre connected to the spectrometer (CompactLine, Avantes BV, The Netherlands). The AvaSoft 8 software (Avantes BV, Apeldoom, The Netherlands) was used to record both real-time spectral characteristics and the time course for defined parameters.

### 4.3. SPR Chip Modification with Graphene

The SPR chip, composed of SF11 glass, coated with 2 nm titanium adhesive layer and 50 nm of gold, was fabricated using e-beam deposition technique (VST e-gun; 1 × 10^−7^ Torr with deposition rate of 1 Å/s). Before graphene transfer, the SPR chip was cleaned by ultrasonication in IPA.

The detailed procedure can be found elsewhere [20]. Single-layer CVD graphene, on 25 µm thick copper foil, was purchased from Graphenea (San Sebastian, Spain). Poly (methyl methacrylate) (350k, Sigma-Aldrich, St. Louis, MI, USA) 2% *w*/*w* solution in THF (99.9%, Carl Roth, Karlsruhe, Germany) was spin-coated (2000 rpm, 1 min) on top of the graphene/copper foil. The backside of the copper foil was treated in HCl (30%):H_2_O_2_ (25%):H_2_O solution (1:2:20). The copper was removed in aqueous solution of ammonium persulfate (0.1 M). The PMMA/graphene film was rinsed in DI and transferred onto the clean gold surface of SPR chip. To flatten the PMMA on the substrate and improve the adhesion of graphene, we annealed the chip at 160 °C for 30 min. Then, the PMMA was removed in an acetone bath. The chip was washed with DI water and dried under nitrogen flow. To evaporate residual water, 30 min annealing at 200 °C was used.

### 4.4. Aptamer Covalent Bonding

Anti-CYN aptamer with the sequence of 5′-CCGATGGTCCGGCCACCCTAAC-AACCAGCCCACCCA-3′ [22] with an amino-modified 5′ end and purified by HPLC was purchased from Metabion AG (Planegg, Germany) and dissolved in 1 × PBS to 3 μM solution. The working concentration was prepared in 1 × PBS containing 1 mM MgCl_2_. To activate the graphene surface, the SPR/graphene chip was soaked in PBASE 1 mM solution in dimethylformamide (DMF) for 3 h. Then, chips were washed in IPA and DI consequently for 3 min each and dried with nitrogen. *N*-hydroxysuccinimide cross-linking reaction was used to bind anti-CYN aptamer covalently. The process was carried out overnight in a humid atmosphere. The unreacted aptamer was removed by shaking in PBS bath.

### 4.5. Cylindrospermopsin Solution Preparation and Measurements

CYN solution in water (10 μg/mL) was purchased from Sigma-Aldrich (USA). It was dissolved in 1 × PBS and 0.1 × PBS solutions several times to reach the needed concentration in the range from 1 pg/L to 10 μg/L. Each concentration was added to the sensor surface during the measurements. The experiments were repeated twice with a regeneration step comprising placing the Gr/SPR chip in 4M urea solution. For statistical analysis, the error was calculated either as standard deviation of experimental data or using noise level of the setup.

## Figures and Tables

**Table 1:** Analytical performance of CYN detection methods.

Method	Detection Limit (pg L^−1^)	Dynamic Range (pg L^−1^)	Ref.
EIS based TH–G/GCE	117 × 10^3^	390 × 10^3^–78 × 10^6^	[32]
MBio biosensor	80 × 10^3^	(80–520) × 10^3^	[33]
EIS aptasensor	41 × 10^3^	41 × 10^3^–33 × 10^6^	[30]
UHPLC-MS	100	(25–500) × 10^3^	[34]
SERS	90	-	[35]
Gr/SPR aptasensor	100	-	this work

EIS—Electrochemical Impedance Spectroscopy; TH–G—thionine–graphene; SERS—surface-enhanced Raman spectroscopy, UHPLC-MS—ultra-high-performance liquid chromatography coupled with mass spectrometry.

## Data Availability

The data presented in this study are available on request from the corresponding authors.

## Supplementary Materials

The following supporting information can be downloaded at https://www.mdpi.com/article/10.3390/toxins15050326/s1: Figure S1: Optical images of two Gr/SPR chips. Figure S2: EDX data for Gr/SPR chip: (a) EDS spectrum; (b) Element concentrations. Figure S3: EDX data for SPR chip: (a) EDS spectrum; (b) Element concentrations. Figure S4: Atomic force microscopy of graphene on SPR substrate: (a) Topography; (b) Phase. Figure S5: Time evolution of I_s,max_ change and wavelength shift.
