# Supplementary material for: Label-Free Direct Detection of Cylindrospermopsin via Graphene-Enhanced Surface Plasmon Resonance Aptasensor
Source: Toxins (Basel). 2023 May 10;15(5):326. doi: 10.3390/toxins15050326 (PMC10220545; doi:10.3390/toxins15050326)
Supplement: Supplementary file 1 [file toxins-15-00326-s001.zip › toxins-2328941-supplementary.pdf]

Visualization of graphene on the gold film

The visualization of monolayer graphene on gold (or other metallic) substrates is a challenging task in comparison to visualization of graphene on Si/SiO_2, where the interference effect increases the effective absorption of graphene up to 15% [1]. Unfortunately, in the case of metallic, especially plasmon active substrates, the contrast between graphene and metal is very low, limiting the application and processing in such combinations. On the other hand, unlike the optical properties, the chemical and surface properties of graphene and metal are different [2]. In this work, we used energy surface difference to highlight variations in wettability to observe graphene by naked eye. The SPR chips were placed in a chamber with high humidity and closed using parafilm. After holding for 5 min at room temperature, the chamber was placed in a refrigerator at 3 °C for 2 min. After the cover was wetted, it was removed, and graphene was visualized (Figure S1) before condensed water evaporated.

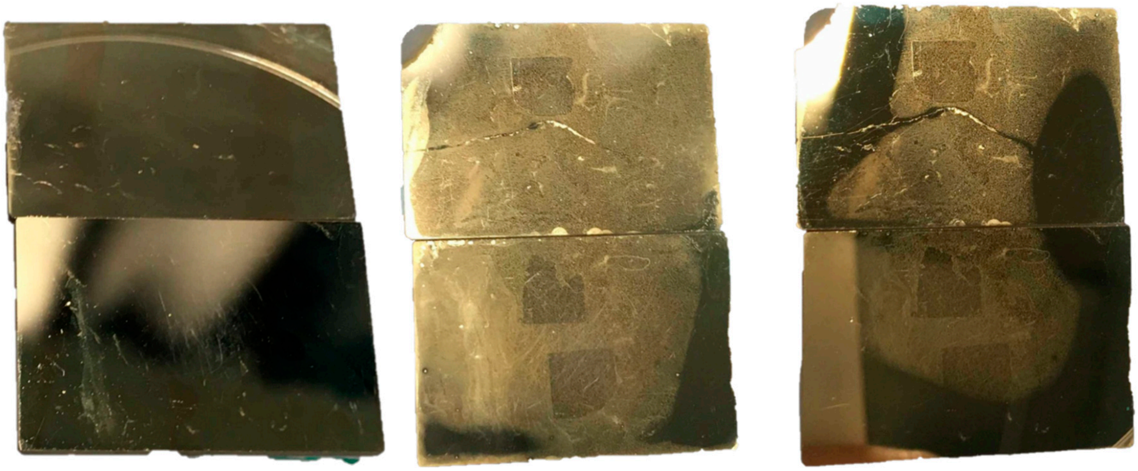

a b c

Figure S1. Optical images of two Gr/SPR chips with one (top) and two (bottom) graphene structures before water vapors condensation (a), and after 1 min (b) and 2 min (c) after opening humid chamber to air.

EDX analysis

EDX analysis of Gr/SPR (Figure S2) and pristine SPR (Figure S3)

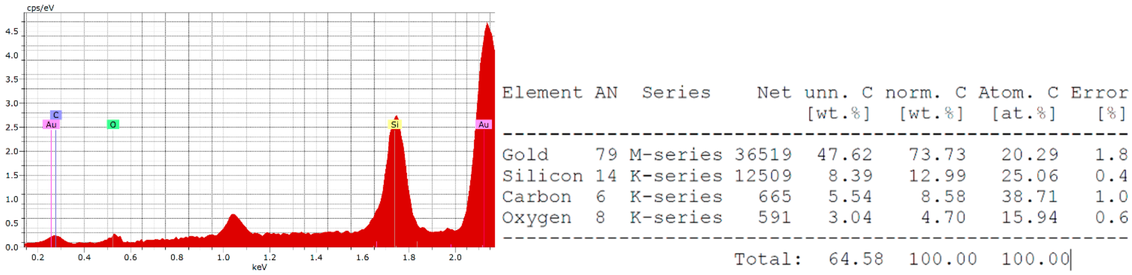

Element	AN	Series	Net	unn. C [wt.%]	norm. C [wt.%]	Atom. C [at.%]	Error [%]
Gold	79	M-series	36519	47.62	73.73	20.29	1.8
Silicon	14	K-series	12509	8.39	12.99	25.06	0.4
Carbon	6	K-series	665	5.54	8.58	38.71	1.0
Oxygen	8	K-series	591	3.04	4.70	15.94	0.6
			Total:	64.58	100.00	100.00	

a b

Figure S2. EDX data for Gr/SPR chip: (a) EDX spectrum; (b) Element concentrations.

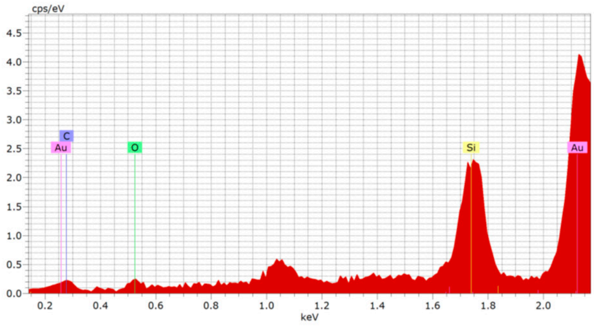

Element	AN	Series	Net	unn. C [wt.%]	norm. C [wt.%]	Atom. C [at.%]	Error [%]
Gold	79	M-series	9176	44.97	74.80	20.97	1.7
Silicon	14	K-series	3006	7.13	11.86	23.32	0.3
Carbon	6	K-series	164	5.07	8.44	38.81	1.3
Oxygen	8	K-series	153	2.94	4.90	16.90	0.8
			Total:	60.12	100.00	100.00	

a b

Figure S3. EDX data for SPR chip: (a) EDX spectrum; (b) Element concentrations.

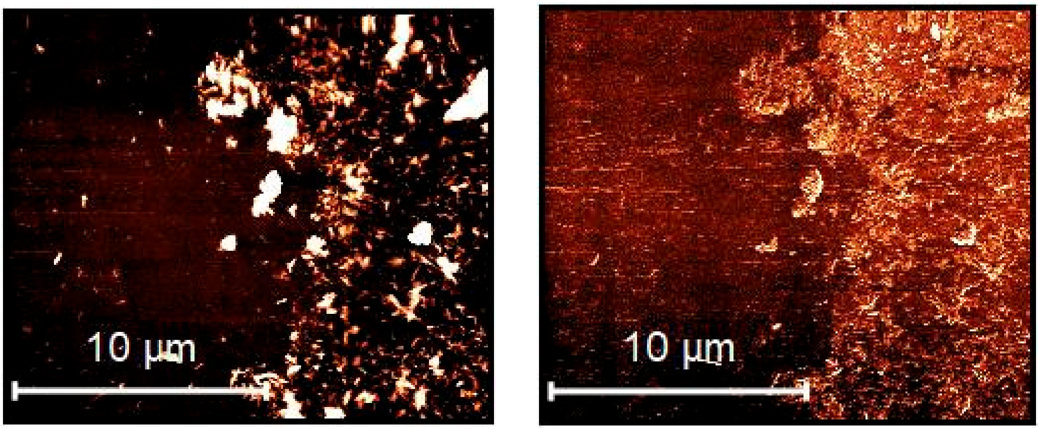

a b

Figure S4. Atomic-force microscopy of graphene on SPR substrate. Topography (a) and phase shift contrast (b) of graphene edge on gold surface. Average roughness of gold film and graphene on gold are 3.5 nm and 9 nm, respectively.

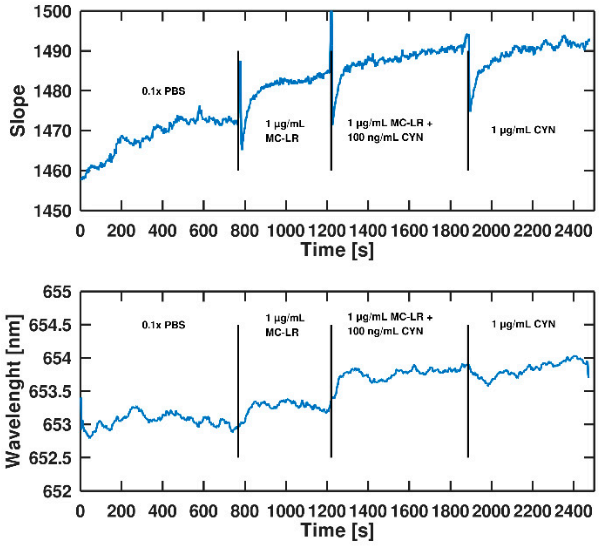

Figure S5. Time evolution of $I_{s,max}$ change (top) and wavelength shift (bottom) regenerated in urea Gr/SPR chip for consequential replacement 0.1xPBS by 1 µg/mL solution of MC-LR, its mixture with 0.1 µg/mL of CYN, and pure 1 µg/mL of CYN.
